# Navigating the Commonality of Healthcare Failures: COVID-19 and Conflict Zones

**DOI:** 10.7759/cureus.50668

**Published:** 2023-12-17

**Authors:** Shampa Ghosh, Manchala Raghunath, Jitendra K Sinha

**Affiliations:** 1 Biomedical Sciences, GloNeuro, Noida, IND; 2 Endocrinology and Metabolism, Indian Council of Medical Research, Hyderabad, IND

**Keywords:** global health challenges, crisis management, world health organization (who), united nations (un), conflict zones, covid-19, healthcare failures

## Abstract

The weaknesses of healthcare systems have been sharply revealed amid the instability of the COVID-19 pandemic and the ongoing conflicts across the borders of different countries. One thing unites these two crises that appear to be separate: the incapacity of healthcare systems to provide for the most basic human requirements in emergency situations. With an emphasis on the roles of the United Nations and the World Health Organisation, we look into the similarities between healthcare failures in COVID-19 and conflict zones in this Editorial and offer possible solutions to improve the circumstances.

## Editorial

Amidst the turbulence of the COVID-19 pandemic and the enduring conflicts such as that presently happening between Palestine and Israel, the vulnerabilities of healthcare systems have been starkly exposed. These two seemingly distinct crises share a common thread: the inadequacies of healthcare systems to meet the most fundamental human needs during times of crisis. In this perspective article, we explore the parallels between healthcare failures in COVID-19 and conflict zones and suggest ways to improve the situations, with a focus on the roles of the United Nations (UN) and the WHO.

A global wake-up call on healthcare failures during COVID-19

COVID-19 exposed the shortcomings of robust healthcare systems that were unprepared to handle a pandemic of such magnitude. First, the healthcare systems worldwide were unprepared to handle a new virus and its structural variants during the pandemic due to inadequate reserves of essential medical supplies and insufficient infrastructure to manage the massive surge in patients [1]. Additionally, the pandemic magnified existing healthcare disparities, with marginalized communities bearing the brunt of the virus's impact. Inadequate access to testing, treatment, and vaccines exacerbated these inequities [2].

Misinformation and a lack of clear, consistent communication undermined public trust and adherence to health guidelines, emphasizing the importance of science communication and public education [3]. Furthermore, the pandemic emphasized the need for global cooperation, as COVID-19 knows no borders. The UN and the WHO played essential roles in coordinating responses and facilitating equitable vaccine distribution, although challenges and inequalities persisted [4].

Healthcare challenges in conflict zones like the Palestine-Israel conflict

In conflict zones, such as the Palestine-Israel conflict, access to healthcare is a rare privilege. The destruction of medical facilities, restrictions on movement, and violence against healthcare workers hinder the delivery of essential services. Conflict-related trauma exacts a heavy toll on mental health, yet mental health services are scarcely available, leaving residents to grapple with profound psychological scars. Moreover, conflicts often result in mass displacement, creating densely populated and unsanitary conditions. This environment becomes a breeding ground for infectious diseases, compounding the already heightened health risks faced by the population. Tragically, violations of international humanitarian law, which is meant to safeguard healthcare workers and facilities in conflict areas, are recurrent issues [5]. This undermines the very principles established to protect healthcare in times of crisis, exacerbating the challenges in providing medical care in these zones of turmoil.

Ways to improve healthcare in crisis situations

Improving healthcare in crisis situations demands a comprehensive approach to address a multitude of challenges. One vital step is to invest in preparedness to ensure that healthcare systems are well-equipped to tackle pandemics and crises. Drawing lessons from the COVID-19 pandemic, governments must allocate resources for critical infrastructure, medical supplies, and surge capacity. This proactive approach serves as a foundation for effective crisis management. Equity and access to healthcare are fundamental principles that must be prioritized, both in the context of COVID-19 and conflict zones. Regardless of socioeconomic status, nationality, or geographic location, every individual should have access to essential healthcare. The UN and the WHO play pivotal roles in advocating for equity and influencing global health policies.

The significance of accurate and transparent science communication cannot be overstated. Governments, international organizations, and healthcare authorities must invest in clear, consistent messaging to build public trust. Effective communication empowers individuals to make informed decisions and adhere to recommended health guidelines. Global cooperation is paramount in crisis situations. The UN and WHO are essential actors in facilitating collaboration among nations. These organizations must continue to coordinate responses, share information, and ensure that resources, including vaccines and medical supplies, are distributed equitably to address the global health challenges that crises present.

The protection of healthcare workers and facilities is non-negotiable. The international community, including the UN, must enforce and strengthen international humanitarian law to safeguard the individuals and infrastructure dedicated to providing healthcare in conflict zones [5]. Mental health services, often overlooked in such environments, are crucial. International organizations can play a pivotal role in advocating for and providing resources to support mental health programs in conflict zones. Acknowledging and addressing the mental health needs of affected populations is a key aspect of comprehensive crisis management. Inclusive decision-making processes in healthcare are indispensable. All stakeholders, including affected communities, should have their voices and perspectives considered during and after crises. This approach ensures that healthcare strategies are tailored to the unique needs of those they serve, promoting more effective and responsive crisis management.

The advancement of mobile health app development would facilitate the swift exchange of medical information between patients and healthcare providers, thereby reducing the necessity for in-person hospital/medical camp visits. Telemedicine also grants individuals access to vital medical information and direct communication with healthcare providers. These lead to the potential alleviation of emergency room congestion, offering a multidisciplinary approach to preliminary healthcare access for both patients and care providers. Additionally, it presents an invaluable means of minimizing physical contact, a vital consideration during pandemics and other infectious outbreaks. However, health apps as well as telemedicine need electricity and mobile phone signal, both of which again are basic needs but not easily available to people in conflict zones.

The Role of the UN and the WHO

The UN and the WHO, as key international organizations, play pivotal roles in responding to global crises. However, their effectiveness in addressing healthcare failures during COVID-19 and in conflict zones has faced scrutiny. The UN, through its agencies like the United Nations Children's Fund (UNICEF) and the World Food Programme, has been at the forefront of humanitarian responses in conflict zones. However, challenges persist, particularly when it comes to political divisions among member states. To improve its effectiveness, the UN should prioritize diplomacy and conflict resolution, emphasizing the protection of healthcare services as a critical aspect of peacebuilding.

The WHO, as the world's leading health authority, has worked tirelessly during the COVID-19 pandemic, guiding countries in their responses and facilitating the equitable distribution of vaccines through initiatives like COVID-19 Vaccines Global Access (COVAX). To enhance its effectiveness, the WHO should continue advocating for vaccine equity, stronger pandemic preparedness, and resilient healthcare systems, while working to strengthen international health regulations. There is still a need for improved preparedness, equity, international cooperation, and incorporation of human rights in conflict zones. The roles of the UN and the WHO are central to addressing these issues. By learning from the past and establishing a collective commitment to healthcare resilience and equity, we can navigate the complexities of healthcare in crisis situations and strive for a world where health is a fundamental human right, irrespective of circumstance.
